# *Ad libitum* feeding of silkworm larvae powder-containing diets specifically influences metabolism-related and short-chain fatty acid-producing gut bacteria in mice

**DOI:** 10.3389/fcimb.2024.1383774

**Published:** 2024-06-14

**Authors:** Aito Murakami, Haruka Yamaguchi, Fu Namai, Takashi Sato, Maki Yamazaki, Hiroshi Uehara, Tadashi Fujii, Takumi Tochio, Kunihiro Shiomi, Takeshi Shimosato

**Affiliations:** ^1^ Department of Biomolecular Innovation, Institute for Biomedical Sciences, Shinshu University, Minamiminowa, Kamiina, Nagano, Japan; ^2^ Food and Feed Immunology Group, Laboratory of Animal Food Function, Graduate School of Agricultural Science, Tohoku University, Sendai, Japan; ^3^ Faculty of Textile Science and Technology, Shinshu University, Ueda, Nagano, Japan; ^4^ Morus Inc., Higashigotanda, Shinagawa-ku, Tokyo, Japan; ^5^ Department of Medical Research on Prebiotics and Probiotics, Fujita Health University, Toyoake, Aichi, Japan

**Keywords:** *Alistipes*, *Clostridium*, *Lachnospiraceae*, microbiota, short-chain fatty acid, silkworm larvae

## Abstract

Silkworm (*Bombyx mori*) larvae are expected to be useful as an ingredient in entomophagy. They are full of nutrients, including indigestible proteins; however, there have been few studies on the effects of the consumption of the entire body of silkworms on the intestinal microflora. We prepared a customized diet containing silkworm larval powder (SLP), and investigated the effects of *ad libitum* feeding of the SLP diet on the intestinal microbiota and the amount of short-chain fatty acids (SCFAs) in mice. We found that the diversity of the cecal and fecal microbiota increased in the mice fed the SLP diet (SLP group), and that the composition of their intestinal microbiota differed from that of the control mice. Furthermore, a genus-level microbiota analysis showed that in the SLP group, the proportions of *Alistipes*, *Lachnospiraceae* A2, and RF39, which are associated with the prevention of obesity, were significantly increased, while the proportions of *Helicobacter* and *Anaerotruncus*, which are associated with obesity, were significantly decreased. Additionally, the level of butyrate was increased in the SLP group, and *Clostridia* UCG 014 and *Lachnospiraceae* FCS020 were found to be associated with the level of butyrate, one of the major SCFAs. These findings indicated that silkworm powder may be useful as an insect food that might also improve obesity.

## 1 Introduction

Silkworms (*Bombyx mori*) are insects belonging to the order Lepidoptera, which includes butterflies and moths. They undergo metamorphosis with four stages of development, i.e., egg, larva, pupa, and adult, within 2 months (Dong et al., 2022). Silkworms were domesticated approximately 5,000 to 10,000 years ago through the breeding of wild silkworms (*Bombyx mandarina*) for the development of the silk industry (Li et al., 2010). The silkworm is the only domesticated insect whose survival is dependent on humans, and is used as an industrial resource in a wide range of fields, including sericulture, and the production of animal proteins as a bioreactor (Zabelina et al., 2015; Marzoli et al., 2022). More recently, silkworm larvae have been considered to be useful as an ingredient in entomophagy. As a food resource, many different types of insects have been shown to have a positive influence on the host gut environment (Stull et al., 2018; Cunha et al., 2023). However, more studies are required to better understand the mechanisms behind these effects as well as the potential health impacts of different types of insects.

Silkworms have several unique characteristics that may make them a particularly useful food resource. For instance, the primary food source of silkworms is mulberry leaves from the family Moraceae; mulberry leaves contain many nutrients, such as sugars, lipids, proteins, minerals, and vitamins, which are essential for silkworm growth (Dong et al., 2017; Qin et al., 2020). Silkworms also have a silk gland lumen in which silk fiber protein is synthesized, secreted, and stored; this silk fiber protein is used in the pupa stage for protecting the silkworm from predators and pathogenic microorganisms (Dong et al., 2016, 2022). Silk fibers, which are essential for the growth of silkworms, are composed mainly of fibroin and sericin, which are non-digestible proteins, *i.e.*, resistant proteins (RPs) (Yuan et al., 2012; Wang et al., 2019; Dong et al., 2020). RPs have physiological effects that help maintain health throughout the digestive tract, and they are broken down by intestinal bacteria (Chen et al., 2016).

Since the whole body of silkworms contains various nutrients, including those in organs (such as silk glands containing RPs), we hypothesized that the consumption of whole silkworm bodies may have beneficial effects on the host gut environment via effects on the intestinal bacteria. Thus, in the present study, we investigated the effects of the oral administration of silkworm larval powder (SLP) on the intestinal microbiota in mice.

## 2 Materials and methods

### 2.1 Silkworm larval powder

Silkworm larvae (3-day-old fifth instar larvae) of the Kosetsu strain of *B. mori* were used. The silkworm larvae were frozen at -27°C, and freeze-dried at -86°C and 4 Pa for 24 h using an FDM-2000 freeze-dryer (EYELA, Tokyo, Japan). Then, 3 to 5 g of silkworm and metal cones were added to 50-mL crush tubes, and the silkworms were crushed at 3,000 rpm for 20 to 30 s using a Multi-beads Shocker device (Yasui Instruments Co., Ltd., Osaka, Japan). The resulting powder was used as the SLP in the following experiments.

### 2.2 *In vivo* mouse study

Female C57BL/6 mice (5 weeks old) were purchased from SLC Japan, Inc. (Shizuoka, Japan), and kept in plastic cages under a 12-h light/dark cycle in a room with a constant and controlled temperature (24°C ± 1°C). Mice were acclimatized with free access to MF feed (Oriental Yeast Co., Tokyo, Japan) and sterile distilled water for 1 week. After the acclimatization period, the mice were randomly divided into a control diet group (Ctrl group) and an SLP diet group (SLP group). The SLP group was fed an SLP-containing diet consisting of 95% AIN-93G and 5% SLP, and the Ctrl group was fed only AIN-93G for 4 weeks (Oriental Yeast Co., Ltd., Tokyo, Japan). Both groups were provided sterile distilled water as drinking water, and had *ad libitum* access to their respective diets and water. The body weight was measured once a week, and the water intake and food intake were measured twice weekly. After the feeding period, the mice were euthanized by cervical dislocation, and their cecal and fecal contents were collected. The weight of each sample was determined using an electronic balance (SEFI IBA-200 Calibration, Micro Precision Calibration Inc., Grass Valley, CA, USA).

### 2.3 16S rRNA gene sequencing

DNA was extracted from the cecal and fecal content samples using a fecal collection kit, and the 16S rRNA gene (V3-V4 region) was amplified according to the methods of a previous report (Takahashi et al., 2023). The constructed DNA was sequenced using the Illumina MiSeq platform (Illumina, San Diego, CA, USA) and a MiSeq Reagent Kit v3 (Illumina). Microbiota analysis was performed using the sequence analysis results from the Quantitative Insights Into Microbial Ecology version 2 (Qiime2) bacterial flora analysis software (Bolyen et al., 2019). The α-diversity, β-diversity, and changes in the intestinal flora at the phylum and genus levels were analyzed from the resulting qzv files using Qiime2view. The α-diversity was analyzed using the observed amplicon sequence variants (ASVs), Faith’s phylogenetic diversity (PD), and Shannon diversity. The β-diversity was analyzed using the Bray-Curtis dissimilarity. In addition, multiple comparison tests using the Tukey-Kramer method were performed to analyze the intestinal bacteria that differed significantly between the SLP and Crtl groups at the genus level.

### 2.4 HPLC analysis of butyrate in the cecal contents

The amount of butyrate in each cecal content sample was measured according to the methods of a previous report (Takahashi et al., 2023). Briefly, a 20- to 50-mg cecal content sample was vortexed for 30 s in phosphate-buffered saline (PBS). After centrifugation at 15,000 rpm and 4°C for 2 min, the supernatant was used for the labeling reaction. Fifty microliters of the supernatant was mixed with 50 μL of PBS, 200 μL of 2 mM caproic acid (Fujifilm Wako, Osaka, Japan), 200 µL of 20 mM 2-nitrophenylhydrazine (in water), and 200 µL of 0.25 M N-(3-dimethylaminopropyl)-N′-ethylcarbodiimide hydrochloride HCl [in ethanol, with an equal volume of 3% pyridine in ethanol (v:v)], then heated at 60°C for 20 min. Subsequently, 200 µL of 15% (w/v) potassium hydroxide was added, and the mixture was incubated at 60°C for 15 min. The reaction mixture was added to 4 mL of a 3.8:0.4 (v:v) mixture of 0.03 M PBS (pH 6.4):0.5 M hydrochloric acid, then filtered through a 0.45-µm filter (PTFE, Puradisc™ 13 mm Syringe Filters, Whatman, Kent, England). The butyrate derivatives were extracted using 5 mL of diethyl ether. The diethyl ether layer was evaporated to dryness under a nitrogen stream at room temperature. The residue was dissolved in 200 µL of methanol, and a 10-µL sample was analyzed by high-performance liquid chromatography (HPLC) to quantify the butyrate.

### 2.5 Statistical analysis

Statistical analysis was performed using Prism software (version 7; GraphPad Software, San Diego, CA, USA). Outliers were identified using the ROUT method (Q = 2%), and were omitted prior to further statistical analyses. Statistically significant differences were assessed by two-tailed ordinary one-way analysis of variance followed by Tukey’s multiple comparisons tests when statistical significance (p < 0.05) was indicated. Results are presented as the mean ± standard error (SE). Spearman’s rank correlation coefficient was calculated for correlation analyses, and the p-value was derived from the Spearman’s rank correlation coefficient.

## 3 Results

### 3.1 SLP-feeding experiment

**Figure 1:**
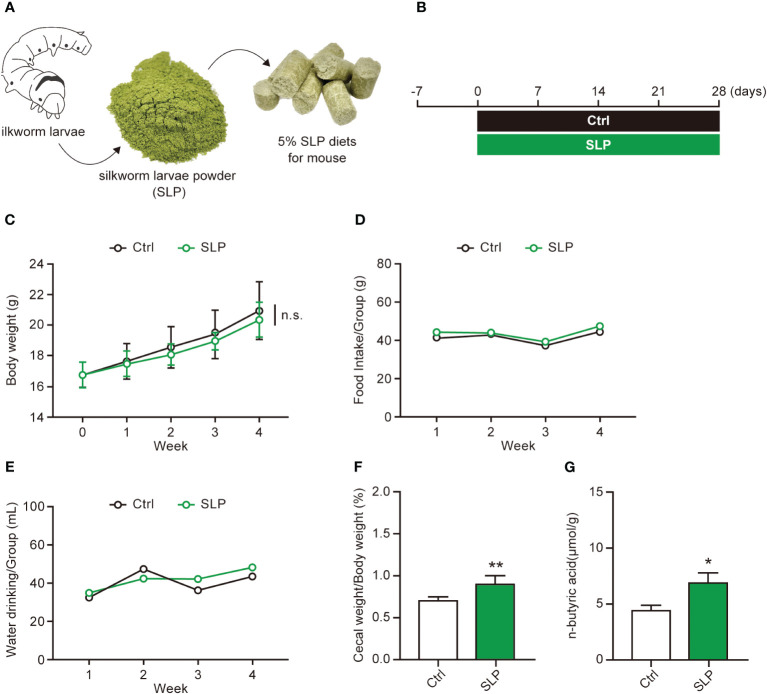
Effects of the SLP diet on the cecal contents and n-butyric acid level. **(A)** Schematic of the preparation of the SLP diet. **(B)** The experimental timeline for examining the effects of *ad libitum* feeding of the SLP diet in mice. **(C-E)** The body weight was measured every week, and the food intake and water intake were measured twice weekly during the experimental period (*n* = 13 per group). **(F)** The ratio of the cecal weight to the body weight after the cecum was collected from each mouse on the final experimental day (*n* = 13 per group). **(G)** The n-butyric acid levels in the cecal contents as measured by HPLC (*n* = 10 per group). Data are shown as the mean ± standard error (SE). *p < 0.05, **p < 0.01.

**Figure 1A** shows a schematic for the preparation of the 5% SLP diet that was fed to the mice. The composition of the SLP diet is shown in **Table 1**, and the experimental timeline is shown in **Figure 1B**. During the experimental period, the body weights steadily increased, and the food and water intakes did not differ significantly between the Ctrl and SLP groups (**Figures 1C-E**). After the mice were euthanized at the end of the experiment, the ratio of the cecal weight to the body weight, and the level of n-butyric acid in the cecal contents as measured by HPLC were significantly higher in the SLP group than in the Ctrl group (**Figures 1F, G**), indicating that the SLP diet affected the microbiota and SCFA production.

**Table 1:** Composition of the silkworm larval powder.

General ingredients	g/100 g
Moisture	3.00
Protein	62.79
Lipid	15.40
Ash	9.50
Carbohydrates	9.31

### 3.2 The SLP diet changed the diversity of the gut microbiota

**Figure 2:**
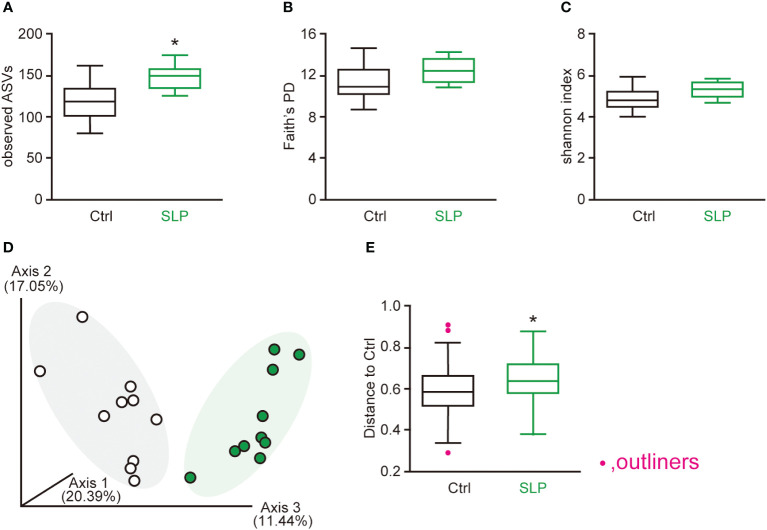
The effects of the SLP diet on the α-diversity and β-diversity in the cecal contents. **(A-C)** The observed number of ASVs **(A)**, Faith’s PD **(B)**, and Shannon index **(C)** were calculated for the two groups. **(D, E)** Principal coordinate analysis plots showing the β-diversity compared between the two groups by Bray-Curtis analysis **(D)** and the β-diversity index **(E)**. Statistical calculations were performed after omitting outliers, and the data are shown as the mean ± SE. *n* = 10 per group. *p < 0.05.

To compare the gut microbiota between the two groups, we examined the α- and β-diversity indices based on the 16s RNA V3-V4 sequences in the cecal contents. For the α-diversity, the number of observed ASVs was significantly higher in the SLP group than in the Ctrl group; in contrast, the Faith’s PD and Shannon indices did not differ significantly between the two groups (**Figures 2A-C**). For the β-diversity, different clusters were seen between the two groups, indicating that the SLP diet changed the composition of the cecal microbiota (**Figures 2D, E**).

### 3.3 The SLP diet changed the composition of the cecal microbiota

**Figure 3:**
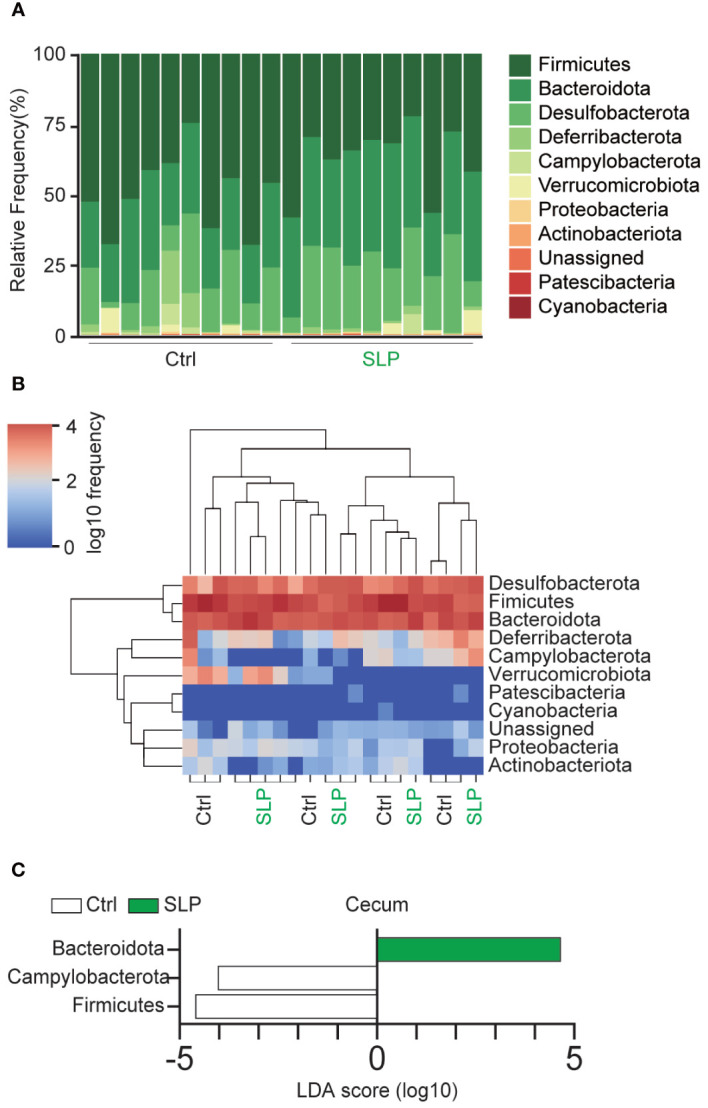
The composition of the gut microbiota in the SLP and Ctrl groups. **(A, B)** A bar plot **(A)** and heatmap **(B)** show the bacterial compositions and frequencies at the phylum level. **(C)** Bacteria with significant differences in the ASVs at the phylum level in the cecal contents between the two groups based on the LDA scores (LDA > 2). ASVs showing significant differences (*p* < 0.05) between the two groups were extracted by the Kruskal-Wallis and Wilcoxon rank-sum tests. *n* = 10 per group.

**Figure 3A** shows a bar plot of the cecal microbiota at the phylum level; the microbiota consisted mainly of Firmicutes, Bacteroidota, Desulfobacterota, and Deferribacterota. A heatmap of the frequency of each microorganism is shown in **Figure 3B**. The frequency of Campylobacterota was lower in the SLP group than in the Ctrl group. The linear discriminant analysis (LDA) scores suggested that the proportions of some bacteria differed significantly between the two groups, i.e., the proportions of Campylobacterota and Firmicutes were significantly higher in the Ctrl group than in the SLP group, and the proportion of Bacteroidota was significantly higher in the SLP group than in the Ctrl group (**Figure 3C**).

### 3.4 Effect of the SLP diet on the gut microbiota at the genus level

**Figure 4:**
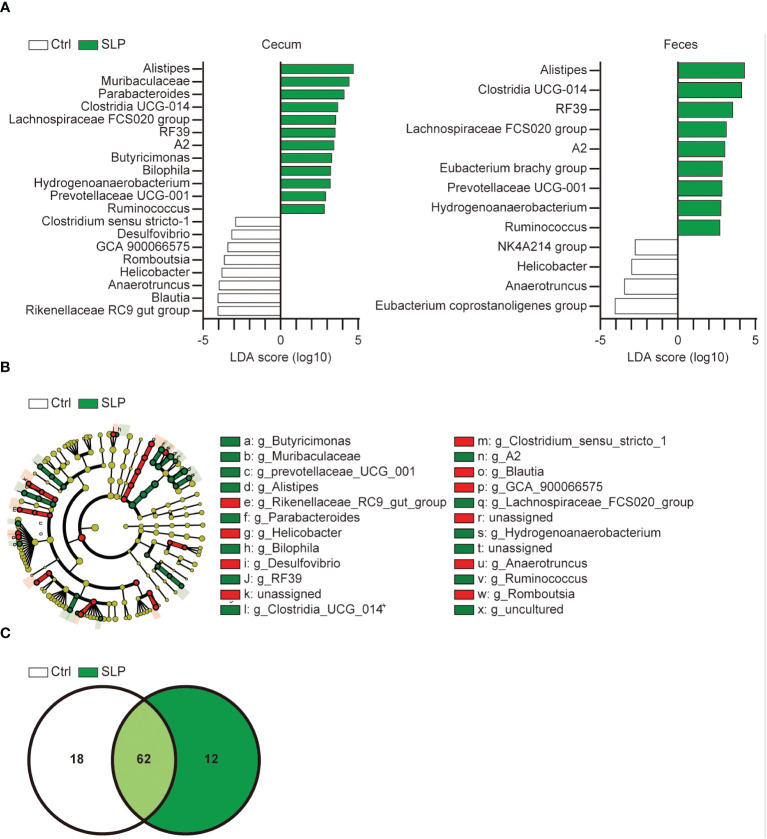
Effect of the SLP diet on the gut microbiota at the genus level. **(A)** Bacteria with significant differences in the ASVs at the genus level in the cecal contents between the two groups based on the LDA scores (LDA > 2). **(B)** A cladogram shows the relationship between taxons in the cecal contents (from the inner to outer rings: phylum, class, order, family, and genus levels). The data were derived from LEfSe analysis (the bar length represents the LDA score). *n* = 10 per group. **(C)** The Venn diagram shows the number of ASVs at the genus level shared between the two groups.

To examine in more detail which bacteria in the cecal and fecal contents were affected by the SLP diet, we further analyzed the LDA scores derived from the LDA effect size (LEfSe) analysis at a lower taxonomic level, *i.e.*, the genus level (**Figure 4A**). Several genera were found in both the SLP and Ctrl groups, including *Alistipes*, *Clostridia* UCG 014, and *Lachnospiraceae* FCS020 group. The cladogram in **Figure 4B** shows that the relationships among specific bacterial clades varied from phylum to genus in each group; there were no remarkable deviations and various bacterial clades were mixed. The Venn diagram in **Figure 4C** shows the number of observed bacterial types at the genus level, and 62 kinds of bacteria are shared between the two groups.

### 3.5 The proportions of representative bacteria affected by the SLP diet

**Figure 5:**
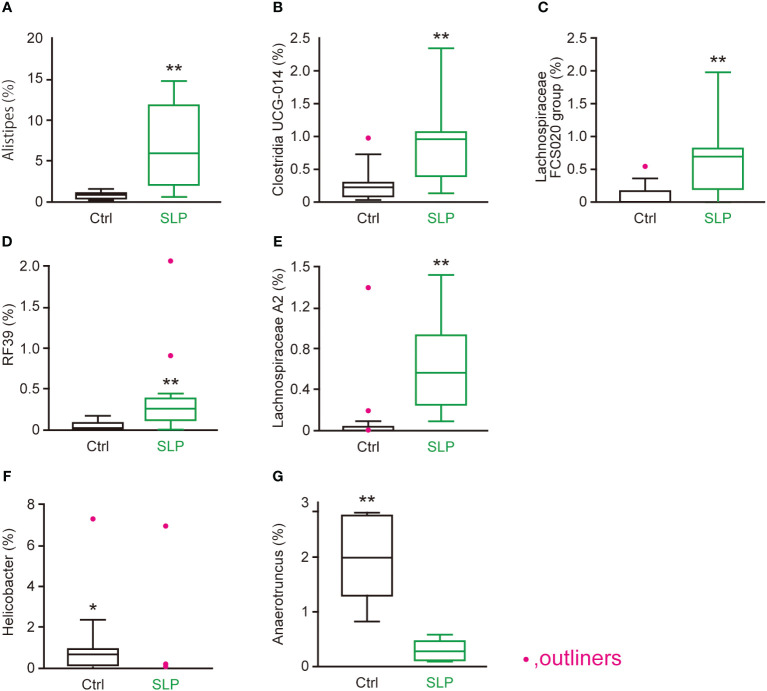
Genera in the cecal contents that were significantly affected by the SLP diet. **(A-G)** The proportions of *Alistipes* **(A)**, *Clostridia UCG-014* **(B)**, *Lachnospiraceae FCS020 group* **(C)**, *RF39* **(D)**, *Lachnospiraceae A2* **(E)**, *Helicobacter* **(F)**, and *Anaerotruncus* **(G)** in the cecal contents as assessed by MiSeq (*n* = 10 each). Statistical analysis was performed after omitting outliers, and the data are shown as the mean ± SE*. n* = 10 per group. *p < 0.05, **p < 0.01.

According to the LDA scores, several microorganisms found in both the cecal and contents were chosen as representative bacteria: *Alistipes, Clostridia* UCG 014*, Lachnospiraceae* FCS020 group, RF39, and *Lachnospiraceae* A2 were chosen as representative bacteria that were significantly more common in the SLP group than in the Ctrl group (**Figures 5A-E**); and *Helicobacter* and *Anaerotruncus* were chosen as representative bacteria that were significantly less common in the SLP group than in the Ctrl group (**Figures 5F, G**). These representative bacteria were further examined in the following analyses.

### 3.6 Correlation analysis of n-butyric acid and the ASVs

**Figure 6:**
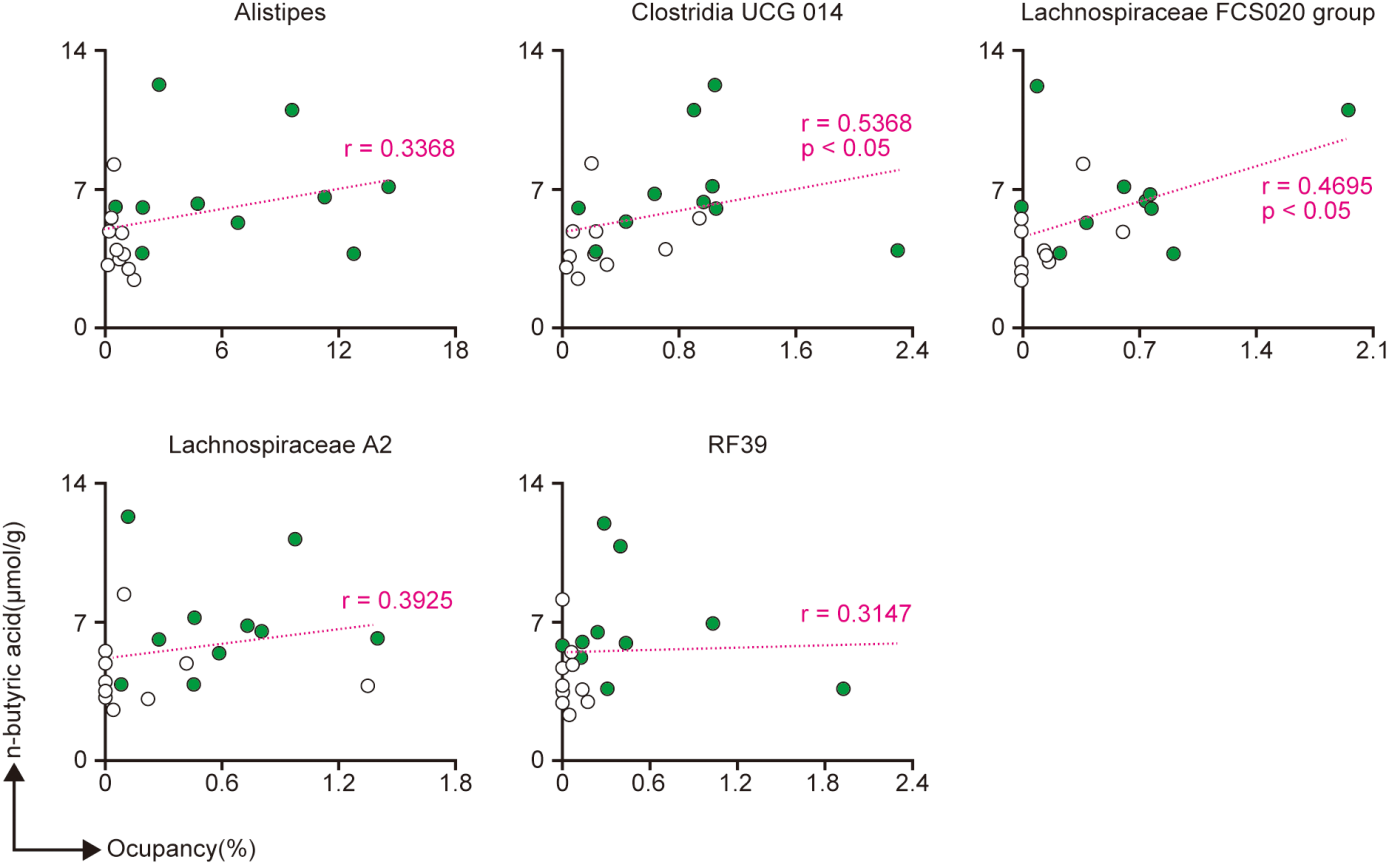
The correlations between the production of n-butyric acid and five genera of bacteria found in the cecal contents. **(A-E)** The correlations between the production of n-butyric acid and the proportions of *Alistipes* **(A)***, Clostridia* UCG 014 **(B)***, Lachnospiraceae* FCS020 group **(C)***, Lachnospiraceae* A2 **(D)**, and *RF39* **(E)**. Spearman’s rank correlation coefficient was used to assess the correlations, and the p-values were derived from the Spearman’s rank correlation coefficient table. *n* = 10 per group.

Since the n-butyric acid level in the cecal contents was higher in the SLP group than in the Ctrl group, we next investigated the relationships between n-butyric acid and the bacteria that were significantly different between the two groups. *Alistipes, Lachnospiraceae* A2 group, and RF39 tended to show a slight positive correlation with n-butyric acid, whereas *Clostridia* UCG 014 and *Lachnospiraceae* FCS020 group showed a strong positive correlation with n-butyric acid (**Figure 6**).

## 4 Discussion

Silkworm larvae, which are rich in nutrients, are expected to be useful as an ingredient in entomophagy (Mahanta et al., 2023). Some unique characteristics of silkworm larvae are that they eat mulberry leaves, and they have silk glands. It is well-known that RPs, which are found in silk fibers, can be utilized by gut microorganisms, and they may have several beneficial effects, *e.g.*, they may protect and help maintain the intestinal environment (Warman et al., 2022). There have been a few reports on the effects of fibroin and sericin derived from the cocoons of silkworm larvae on the gut microbiota and some diseases (Park et al., 2020; Xu et al., 2021; Zhou et al., 2023). However, until now, there have been no studies on the effects of the ingestion of the entire body of silkworm larvae, which contain RPs.

The SLP that we independently prepared contained the entire body of the silkworm larvae, and it thus included all of the components of the silk glands. Silk glands produce and secrete liquid silk, which becomes silk threads (Radtke, 2016). In our study, the SLP diet had no influence on the body weight of the mice, or their food and water intake. However, the cecal weight and n-butyric acid level in the cecal contents were higher in the SLP group than in the Ctrl group, indicating that the SLP diet exerted significant effects (**Figure 1**). N-butyric acid is an SCFA, and SCFAs are known to be metabolites of RPs in gut microorganisms. SCFAs function in maintaining host gut homeostasis (Liu et al., 2021). Ohira et al. (Ohira et al., 2013) found that butyrate can inhibit the production of the inflammatory cytokines; tumor necrosis factor-α, monocyte chemoattractant protein-1, and interleukin-6 in macrophages. In addition, there have been many reports of the protective effect of butyrate on intestinal barrier function (Ma et al., 2012; Wang et al., 2012; Zheng et al., 2017). The SLP diet likely regulates the intestinal environment by increasing the level of n-butyric acid.

To investigate how the gut microbiota changed and which bacteria were involved, we conducted a Qiime2 analysis based on the 16sRNA V3-V4 sequences in the cecal and fecal contents. No differences were seen in the Shannon index between the two groups, but the observed ASVs were significantly higher in the SLP group than in the Ctrl group, and the β-diversity analysis revealed different clusters between the SLP and Ctrl groups. Although the SLP diet had little influence on the species diversity within a group, it modified the structure of the bacterial clusters between two groups (**Figure 2**). The bar plot in **Figure 3A** shows the composition of the gut microbiota at the phylum level, revealing that in both groups, the gut microbiota mainly consisted of bacteria from four phyla, *i.e.*, Firmicutes, Bacteroidota, Desulfobacterota, and Deferribacterota. Of note, *Campylobacter* and *Helicobacter*, which belong to the phylum Campylobacterota (previously called Epsilonproteobacteria), are known to be pathogenic in humans and to inhabit the digestive tract (Beeby, 2015; van der Stel and Wosten, 2019), and our results indicated that the administration of an SLP diet may decrease the amount of Campylobacterota species in the gut (**Figure 3**). In addition, the LDA scores at the genus level revealed that *Helicobacter* and *Anaerotruncus* were among the bacteria that markedly declined in the fecal and cecal contents of mice in the SLP group (**Figures 4**, **5**). *Helicobacter* infection is closely associated with the gut microbiota and various diseases, such as obesity, inflammatory bowel disease, and diabetes (Pellicano et al., 2020). Additionally, *Helicobacter* has a reverse relationship with *Alistipes* (Chen et al., 2021), which was also found at a high frequency in the cecal and fecal contents (**Figures 4**, **5**). According to previous reports, *Anaerotruncus* has a strong positive correlation with obesity-related indices (Liu et al., 2022); in contrast, *Alistipes* is negatively correlated with obesity indices (Dai et al., 2022). Zhao et al. (2022) showed that the administration of adzuki beans effectively decreased the abundance of *Helicobacter*, and altered the gut microbiota to a composition that suppressed high-fat diet-induced body weight gain. These findings strongly suggest that SLP may have the potential to not only suppress the growth of pathogenic bacteria, but also to promote the growth of obesity-suppressing bacteria in the gut microbiota.

Some of the other highly modified bacteria were *Clostridia* UCG 014*, Lachnospiraceae* FCS020 group, RF39, and *Lachnospiraceae* A2. *Clostridia* UCG 014 and *Lachnospiraceae* have been reported to be associated with the production of SCFAs (Vacca et al., 2020; Liu et al., 2023). Similarly, we also found that *Clostridia* UCG 014 and *Lachnospiraceae* FCS020 group were significantly correlated with n-butyric acid, while *Lachnospiraceae* A2 was not (**Figure 6**). Of note, *Lachnospiraceae* A2 has been reported to be correlated with an increase in anti-inflammatory regulatory T cells in the mesenteric lymph nodes that was associated with obesity (Cao et al., 2021; van de Wouw et al., 2021). In other words, *Lachnospiraceae* A2 might improve obesity by regulating inflammation via the induction of regulatory T cells, and by cooperating with the abovementioned obesity-suppressing bacteria. In a study by Dai et al (Dai et al., 2023), the abundance of RF39 was increased by the administration of sodium butyrate to broiler chickens. In our study, the amount of n-butyric acid was increased due to some SCFA-producing bacteria (**Figures 1**, **6**). Namely, the abundance of RF39 may have increased due to stimulation by the n-butyric acid produced by *Clostridia* UCG 014 and *Lachnospiraceae* FCS020 group. Furthermore, Wang et al. (Wang et al., 2020) found that RF39 was prevalent in the gut microbiota, and that it might produce metabolites, such as acetate and hydrogen, that affect the amount of acetic acid, which is an SCFA, in the gut.

In conclusion, by analyzing the changes in the gut microbiota that resulted from the feeding of the SLP-containing diet in mice, we found that the administration of the SLP diet may have several benefits. The SLP diet changed the composition of the gut microbiota; in particular, it reduced the amounts of *Helicobacter* and *Anaerotruncus*, which are associated with obesity, and increased the amounts of *Alistipes*, which are associated with the prevention of obesity and the production of SCFAs. *Clostridia* UCG 014 and *Lachnospiraceae* FCS020 group showed a close association with n-butyric acid, the levels of which were increased in the cecal contents of the SLP group, and the SCFAs produced by these bacteria might promote the growth of RF39. Moreover, the proportion of *Lachnospiraceae* A2, which may potentially reduce inflammation, was high in the SLP group, and it was thought that it might regulate obesity by maintaining the intestinal environment. These results suggested that SLP might help prevent obesity. Nonetheless, more studies are needed in the future to examine its association with obesity, the underlying mechanisms, and other related factors, including studies on the liver, adipose tissues, and responses to insulin and glucose in animal models of obesity. We hope SLP will be useful as a food ingredient that can promote our health by promoting the maintenance of a healthy gut environment.

## Data availability statement

The datasets presented in this study can be found in online repositories. The names of the repository/repositories and accession number(s) can be found below: https://www.ddbj.nig.ac.jp/, PRJDB17364.

## Ethics statement

All experimental procedures were carried out in accordance with the Regulations for Animal Experimentation of Shinshu University. All experimental methods were reviewed by the Committee for Animal Experiments of Shinshu University, and complied with national regulations and guidelines, as specified by Law No. 105 and Notification No. 6 in Japan. The Committee for Animal Experiments of Shinshu University approved the animal protocol (Approval No. 021082). The studies were conducted in accordance with the local legislation and institutional requirements. Written informed consent was obtained from the owners for the participation of their animals in this study.

## Author contributions

AM: Writing – original draft, Writing – review & editing, Investigation, Data curation, Validation, Visualization. HY: Writing – original draft, Investigation, Data curation, Validation. FN: Writing – original draft, Writing – review & editing, Investigation, Data curation. TSa: Writing – original draft, Writing – review & editing, Methodology. MY: Writing – review & editing, Resources. HU: Writing – review & editing, Resources. TF: Writing – review & editing, Investigation, Data curation. TT: Writing – review & editing, Investigation, Data curation. KS: Writing – original draft, Writing – review & editing, Project administration, Conceptualization, Resources. TSh: Writing – original draft, Writing – review & editing, Visualization, Methodology, Funding acquisition, Project administration, Supervision.
